# Global country-level network analysis of mental disorders

**DOI:** 10.1017/gmh.2026.10244

**Published:** 2026-07-20

**Authors:** Mohammadreza Balooch Hasankhani, Shoboo Rahmati, Mark J.M. Sullman, Tania Dehesh

**Affiliations:** 1 Student Research Committee, Kerman University of Medical Sciences, Kerman, Islamic Republic of Iran; 2 Social Determinants of Health Research Center, Research Institute for Health Development, Kurdistan University of Medical Sciences, Sanandaj, Islamic Republic of Iran; 3 Department of Social Sciences, School of Humanities and Social Sciences, University of Nicosia, Nicosia, Cyprus; 4 Department of Biostatistics and Epidemiology, School of Public Health, Kerman University of Medical Sciences, Kerman, Islamic Republic of Iran

**Keywords:** mental disorders, network analysis, ecological study, incidence, Global Burden of Disease

## Abstract

Mental disorders are major contributors to the global burden of disease and may show correlated incidence patterns across countries. We characterized the country-level ecological network of age-standardized incidence rates for 10 mental disorders, identified statistically central disorders and examined how individual countries influenced estimated network connectivity. Data for 204 countries and territories, including Sociodemographic Index values, were obtained from the Global Burden of Disease Study 2023. A regularized network model using the mgm framework estimated conditional associations among country-level variables. Strength centrality and case-deletion delta-centrality analyses assessed disorder connectedness and country influence, with bootstrap procedures evaluating stability. Approximately 400 million incident cases of the studied disorders were estimated globally in 2023. Anorexia nervosa and bipolar disorder had the highest strength centrality, indicating broader conditional connections with other disorders. Australia, the United States, Iran, China and Finland showed relatively large delta-centrality values, suggesting greater influence on estimated centrality than most countries. Strength centrality stability was acceptable but modest. These findings provide a descriptive, hypothesis-generating view of how modeled incidence rates covary across countries and should not be interpreted as individual-level comorbidity, causal influence or direct policy priority.

## Graphical abstract

**Figure:**
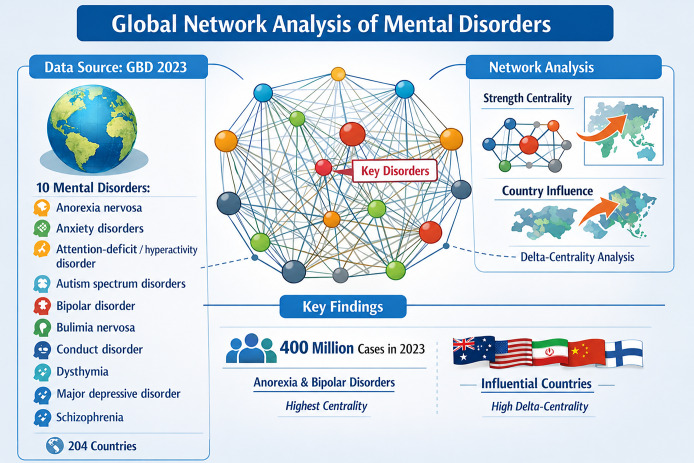

## Impact statement

Mental disorders account for a substantial share of ill health globally, yet cross-country patterns in their incidence are rarely examined jointly. Using GBD 2023 country-level estimates, we mapped a regularized ecological network of 10 mental disorders and the sociodemographic Index across 204 countries and territories. In this context, centrality refers to how strongly a disorder’s national incidence pattern is statistically connected to other nodes in the network after adjustment for the remaining variables, not to how common, severe or clinically important the disorder is. Anorexia nervosa and bipolar disorder had the highest strength centrality, although this centrality should be interpreted cautiously because stability was modest. Countries with large delta-centrality values were those whose exclusion most changed these estimated centrality values, indicating influence on the all-countries network rather than causal importance. This study is descriptive and hypothesis-generating. It may help identify disorders and settings for more detailed comparative, individual-level and longitudinal research, but it does not by itself establish intervention targets or policy priorities.

## Introduction

Mental disorders remain major contributors to global disability and population health loss. Recent Global Burden of Disease estimates continue to show that mental disorders account for a substantial share of years lived with disability worldwide, underscoring the need for coordinated public health, research and health-system responses (Global Burden of Disease Collaborative Network, 2024, 2025). Given the complexity and frequent overlap among these conditions, there is a continuing need for approaches that can characterize broader patterns across disorders and across settings.

Mental disorders often co-occur in the same individuals, leading to complex patterns of comorbidity among conditions such as depression, anxiety, bipolar disorder and substance use disorders (Olfson et al., 2017). These comorbidities have important clinical implications because patients with multiple co-occurring disorders typically experience more severe functional impairments, poorer treatment response and an elevated risk of suicide (Andreescu et al., 2007; Furukawa et al., 2018).

Traditionally, the study of comorbidity has relied on the Common Cause Model, which conceptualizes mental disorders as independent entities and explains symptom overlap by a shared latent cause (Cramer et al., 2010; Caspi et al., 2014). However, accumulating evidence indicates that this approach does not fully account for the overlap and complex interactions among symptoms and may overlook functional connections between them (Borsboom, 2017). In contrast, the network theory of mental disorders views psychopathology as a system of mutually interacting problems that can reinforce one another and contribute to the development and maintenance of comorbidity (Borsboom and Cramer, 2013). This framework has mainly been applied to individual-level data at the symptom or disorder level and enables the identification of statistically central and potentially influential nodes within a network, which is relevant for formulating targeted and preventive interventions.

Most work within the psychopathology network framework has focused on individuals, using clinical samples or population-based surveys to examine relations among symptoms or diagnoses (Fried et al., 2017; Robinaugh et al., 2020). However, patterns of mental disorders can also be studied at higher levels of aggregation. By applying network analysis to country-level data, it is possible to investigate how incidence rates of different mental disorders covary across countries after accounting for sociodemographic context (Roux, 2002). In this setting, network nodes represent country-level incidence rates, and network edges represent associations between these rates across countries, rather than relationships between individual symptoms or diagnoses (Cramer et al., 2010; Borsboom, 2017). Such ecological analyses do not assess comorbidity within individuals and cannot be interpreted causally, but they can reveal cross-national patterns of association, identify disorders that are centrally positioned within these patterns and generate hypotheses about shared determinants or contextual factors (Morgenstern, 1995; Robinaugh et al., 2020).

It is important to distinguish ecological covariation from individual-level comorbidity. In the present study, nodes do not represent patients, symptoms or diagnostic transitions; they represent country-level incidence estimates. Accordingly, observed edges capture cross-national conditional associations in modeled incidence rates after adjustment for the other variables in the network. They do not demonstrate within-person comorbidity, causal mechanisms or treatment targets (Morgenstern, 1995, Robinaugh et al., 2020).

Using GBD 2023 data, we conducted a cross-sectional ecological network analysis of 10 mental disorders across 204 countries and territories, incorporating the Sociodemographic Index (SDI) as a composite measure of development. Our primary aim was to estimate the country-level network of age-standardized incidence rates for these disorders and SDI and identify disorders that occupy relatively central positions in this ecological network. Our secondary aim was to examine how individual countries influenced the estimated centrality of the most connected disorders using a case-deletion delta-centrality approach. Conceptually, the study is informed by network theory, which views psychopathology as an interconnected system, but we extend that logic to the ecological level by examining relationships among country-level incidence patterns rather than among individual symptoms or diagnoses (Borsboom and Cramer, 2013; Borsboom, 2017). The significance of this approach lies in its ability to describe broad global patterning, identify statistically central disorders within country-level incidence structures and highlight settings that may be informative for future comparative, multilevel or longitudinal research. These aims are descriptive and hypothesis-generating and do not involve causal inference or direct identification of intervention targets.

## Methods

### Research design

This was a cross-sectional ecological study based on secondary analysis of publicly available, aggregate country-level estimates from the Global Burden of Disease Study (2023). An ecological design was appropriate because the aim was to examine cross-country co-variation in modeled incidence rates, rather than to assess individual-level comorbidity or causal effects.

### Study units

The analytic units were 204 countries and territories included in GBD 2023. Because the study used aggregate national estimates, there were no individual participants. Throughout the manuscript, countries and territories are therefore treated as study units rather than participants.

### Data source and variable selection

The data for this study were extracted from GBD 2023, produced by the Institute for Health Metrics and Evaluation (IHME) at the University of Washington. This database provides standardized estimates of the burden of diseases, injuries and risk factors for more than 200 countries and territories. We extracted age-standardized incidence rates for 10 disorder categories: anorexia nervosa, anxiety disorders, attention-deficit/hyperactivity disorder, autism spectrum disorders, bipolar disorder, bulimia nervosa, conduct disorder, dysthymia, major depressive disorder and schizophrenia.

These 10 variables were selected to represent a parsimonious set of disorder-level incidence estimates for the present ecological network. In the broader GBD mental disorders framework, burden estimates are produced for 12 mental disorders, with depressive disorders represented by major depressive disorder and dysthymia, and eating disorders represented by anorexia nervosa and bulimia nervosa (Ferrari et al., 2022). In the present study, we retained these four disorder-specific variables, used anxiety disorders as the aggregate GBD category and did not include idiopathic developmental intellectual disability or the residual category of other mental disorders, in order to keep the ecological network focused on disorder categories with clearer cross-national interpretability. Within the IHME GBD methods appendices, anxiety disorders are modeled as “any anxiety disorder” and include panic disorder, agoraphobia, specific phobia, social phobia, obsessive-compulsive disorder, post-traumatic stress disorder, generalized anxiety disorder, separation anxiety disorder and anxiety disorder not otherwise specified; anxiety disorders due to a general medical condition and substance-induced anxiety disorder are excluded (Institute for Health Metrics and Evaluation, 2021).

To account for cross-country heterogeneity in socioeconomic development, we incorporated the SDI into the analysis. SDI is a composite measure derived from three indicators: per capita income, average years of schooling in the population aged 15 years and older and total fertility rate. It reflects the overall level of social and economic development in each country.

All data were extracted at the country level for the year 2023 and organized into a matrix with 204 rows (countries) and 11 columns (10 disorders and SDI). To avoid the influence of differences in measurement scale among variables, all variables were standardized to have a mean of 0 and a standard deviation of 1 before network estimation.

### Statistical analysis

All analyzed variables were continuous country-level measures. Preliminary assessment using Kolmogorov–Smirnov tests indicated significant departures from normality for all variables. We therefore estimated a sparse regularized network using the mgm package in R. In the present application, the mgm framework was used as a flexible regularized estimation approach for continuous ecological variables; no categorical variables were included. Because all variables were continuous and the analysis was ecological, the retained edges are best interpreted as regularized conditional associations between country-level variables after accounting for the remaining nodes in the network. We therefore avoid equating these edges directly with individual-level comorbidity coefficients and interpret them more cautiously than classical partial correlations under strict multivariate normality.

LASSO regularization was applied to reduce spurious edges, and the tuning parameter was selected using the Extended Akaike Information Criterion, consistent with the original analysis. The resulting edge-weight matrix was visualized using the qgraph package. Nodes represented disorder-specific incidence rates or SDI, and edges represented regularized conditional associations between variables given the remaining variables in the model.

We calculated strength centrality as the primary measure of node connectedness in the network. Closeness and betweenness were also computed and are shown for completeness, but because these indices are often less stable in network models, the substantive interpretation in the main text focuses primarily on strength. Disorders with higher strength values were interpreted as being more strongly connected to the estimated ecological network.

To evaluate the stability and accuracy of the network, we conducted nonparametric bootstrapping with 500 resamples using the bootnet package in R. Two types of bootstrap analyses were performed. First, an edge-weight bootstrap was used to compute confidence intervals for each edge and to assess the precision of the estimated relationships between variables. Second, a case-dropping bootstrap was used to examine the stability of node centrality indices. For the latter, the Correlation Stability (CS) coefficient was calculated to quantify the maximum proportion of cases that could be removed while retaining a correlation of at least 0.7 between the original and bootstrapped centrality estimates. CS values above 0.25 are typically interpreted as indicating acceptable stability and values above 0.5 as indicating high stability of the network results (Epskamp et al., 2018). Because the observed CS coefficient for strength centrality was 0.28, centrality rankings were interpreted cautiously.

After identifying the two disorders with the highest strength centrality in the overall network, we addressed the second objective by examining how the omission of each country changed the estimated centrality of these focal disorders. To identify countries whose inclusion had the greatest influence on the estimated connectedness of each focal disorder in the full-sample network, we conducted a country-level case-deletion analysis (Efron and Tibshirani, 1994). First, we estimated the network using all 204 countries and recorded the strength centrality of the focal disorder as the reference value. We then iteratively removed one country at a time from the dataset, re-estimated the network with the same modeling specifications and recalculated the strength centrality of the focal disorder. For each country, delta centrality was defined as the difference between the strength centrality in the full network and the strength centrality after exclusion of that country. This delta value reflects how much the omission of a given country changes the centrality of the focal disorder and, indirectly, its contribution to the overall network structure.

Positive delta values indicate that removing a country reduces the estimated centrality of the focal disorder, suggesting greater influence on the all-countries network estimate. Negative delta values indicate that removing a country increases the estimated centrality of the focal disorder. This case-deletion analysis should be interpreted as an influence diagnostic rather than as evidence of causal importance or intervention priority.

To visualize these patterns, we generated world maps in which countries were colored according to their delta centrality values, using a color scale from green (low or negative delta) to red (high or positive delta). This visualization highlights cross-country heterogeneity in the influence of each country on the ecological network of mental disorder incidence. Countries with relatively large positive delta values were interpreted as countries whose incidence profiles contributed more strongly to the full-sample estimate of the focal disorder’s network connectedness. This does not indicate that the disorder is more clinically central within those countries, but rather that excluding those countries changes the estimated global ecological network. All analyses were conducted using R software (version 4.4.2).

We used publicly available GBD 2023 incidence estimates, conducted the analyses independently of the GBD Collaborator Network and confirmed that this secondary, methodologically focused study does not overlap with any ongoing GBD projects.

## Results

In 2023, there were an estimated 400,412,888 new cases of mental disorders across the 204 countries, corresponding to a global age-standardized incidence rate of 4,862.84 per 100,000 population. Among the studied disorders, major depressive disorder accounted for the largest number of incident cases, with more than 289,610,679 new cases and an age-standardized incidence rate of 3,464.17 per 100,000 population. In contrast, anorexia nervosa had the lowest age-standardized incidence rate, at 16.47 per 100,000 population (Table 1).

**Table 1.** Incidence and age-standardized rates (per 100,000 population) of major mental disorders among world countries, 2023 Table 1. long description.

Cause	Number	Age-standardized rates
Mental disorders	400,412,888	4,862.84 (4,381.09, 5,549.9)*
Anorexia nervosa	1,268,428	16.47 (11.61, 22.51)
Anxiety disorders	55,244,318	684.28 (491.77, 954.63)
Attention-deficit/hyperactivity disorder	4,226,677	59.91 (40.47, 88)
Autism spectrum disorders	1,197,423	19.64 (5.15, 53.64)
Bipolar disorder	2,505,597	30.47 (25.35, 36.13)
Bulimia nervosa	16,957,112	219.08 (136.93, 338.74)
Conduct disorder	13,625,473	182.72 (136.1, 234.05)
Dysthymia	14,094,987	165.73 (136.63, 199.33)
Major depressive disorder	289,610,679	3,464.17 (3,001.81, 3,994.45)
Schizophrenia	1,682,194	20.37 (17.22, 24.05)

* (95% Confidence interval).

**Figure 1.**
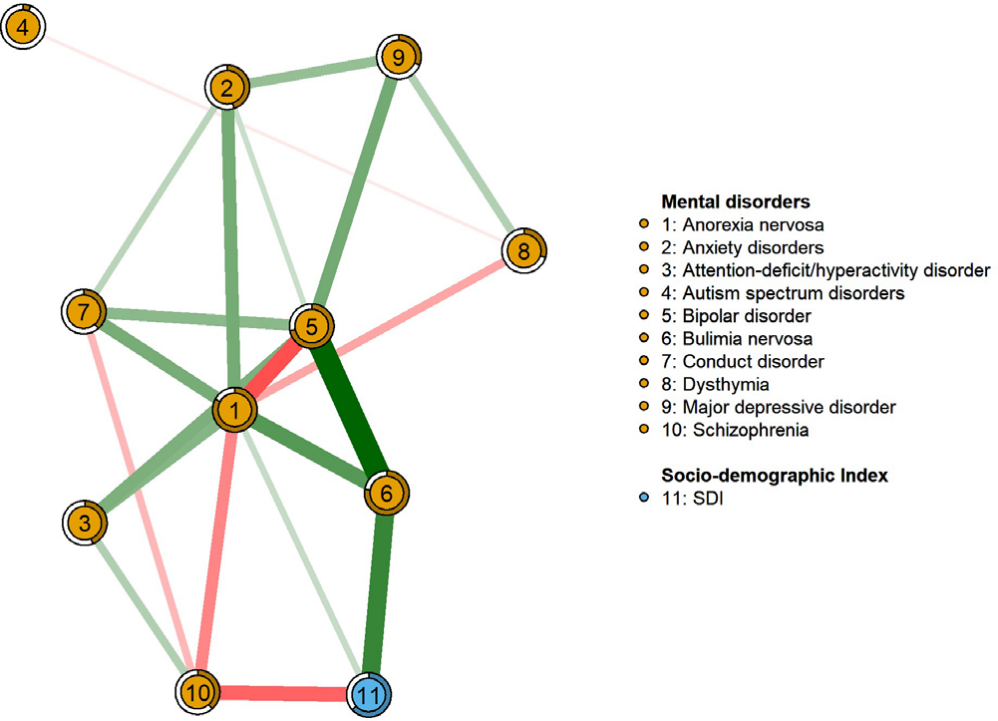
Ecological network of country-level age-standardized incidence rates for 10 mental disorders and SDI across 204 countries and territories, 2023. *Note:* Nodes represent disorder-specific incidence rates or SDI. Node size is shown uniformly for readability and does not encode an additional statistic. Edges represent regularized conditional associations after adjustment for all other nodes in the network. Blue edges indicate positive associations and red edges indicate negative associations. Thicker edges indicate larger absolute edge weights. The figure depicts ecological covariation across countries and should not be interpreted as individual-level comorbidity. Figure 1. long description.

The estimated ecological network of country-level age-standardized incidence rates and SDI in 2023 is shown in Figure 1. After regularization, a subset of pairwise connections was retained as nonzero in the final network. Most retained edges were positive, indicating positive conditional associations between country-level incidence rates after adjustment for the remaining variables in the network. These edges reflect ecological covariation across countries, not individual-level comorbidity.

Because strength was the most interpretable and comparatively more stable centrality metric in this setting, we focus on strength in the main text. Anorexia nervosa had the highest strength centrality (2.26) and eight direct connections, followed by bipolar disorder (1.95) and bulimia nervosa (1.41). Autism spectrum disorders had the lowest strength (0.05) and only one retained edge. Importantly, centrality reflects network connectedness rather than burden. Thus, a disorder can have a low global incidence rate and still be statistically central if its country-level incidence pattern is conditionally associated with multiple other nodes.

**Figure 2.**
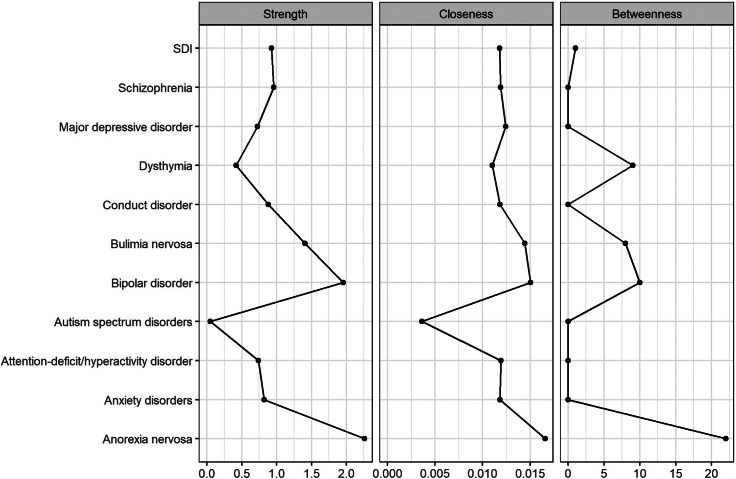
Centrality indices for the ecological network shown in Figure 1. *Note:* Strength was the primary centrality metric used for substantive interpretation in the present study. Closeness and betweenness are displayed for completeness but are interpreted cautiously because the stability of centrality estimates was modest. Figure 2. long description.

For anorexia nervosa, the largest retained absolute edge weights were with bipolar disorder (−0.39), bulimia nervosa (0.38), anxiety disorders (0.31), conduct disorder (0.30) and attention-deficit/hyperactivity disorder (0.27) (Supplementary Table S1). The anorexia nervosa-bipolar disorder edge was negative, whereas the anorexia nervosa-bulimia nervosa edge was positive. Accordingly, the network identifies both shared and inverse ecological associations after conditioning on the other variables. Closeness and betweenness are shown in Figure 2 for completeness but are interpreted cautiously given the modest stability of centrality estimates.

Several negative edges were retained in the regularized network, including anorexia nervosa with bipolar disorder (−0.39), anorexia nervosa with schizophrenia (−0.27), anorexia nervosa with dysthymia (−0.20), conduct disorder with schizophrenia (−0.16), autism spectrum disorders with dysthymia (−0.05) and schizophrenia with SDI (−0.35). These negative conditional associations should be interpreted cautiously. They indicate inverse ecological associations after adjustment for the other nodes in the network, but they do not imply inverse individual-level comorbidity and may be sensitive to model specification and regularization.

**Figure 3.**
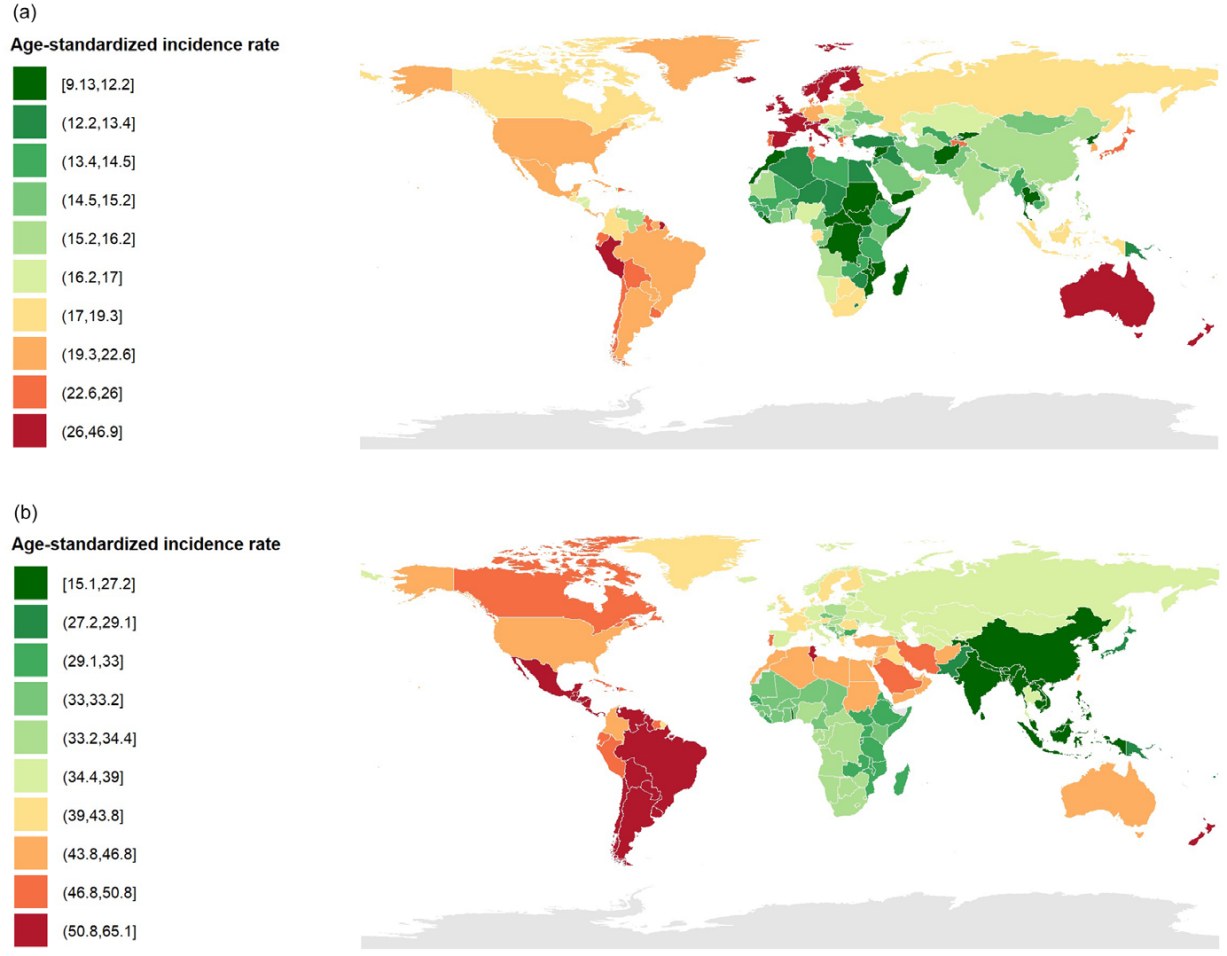
Country-level distribution of age-standardized incidence rates for anorexia nervosa (A) and bipolar disorder (B) in 2023. Figure 3. long description.

Given that anorexia nervosa and bipolar disorder showed the highest strength centrality, country-level maps of their age-standardized incidence rates in 2023 were generated to illustrate their geographic distribution (Figure 3). For anorexia nervosa, higher incidence rates were observed in countries with higher levels of sociodemographic development and in specific geographical regions. The highest rates were seen in Australia, Spain, Finland, Norway and Monaco, while the lowest were observed in Afghanistan, Yemen, Somalia, Sudan and Burundi. Bipolar disorder showed the highest incidence rates in New Zealand, Mexico, Brazil, Paraguay and Argentina and the lowest rates in China, Tajikistan, North Korea, Timor-Leste and Togo.

**Figure 4.**
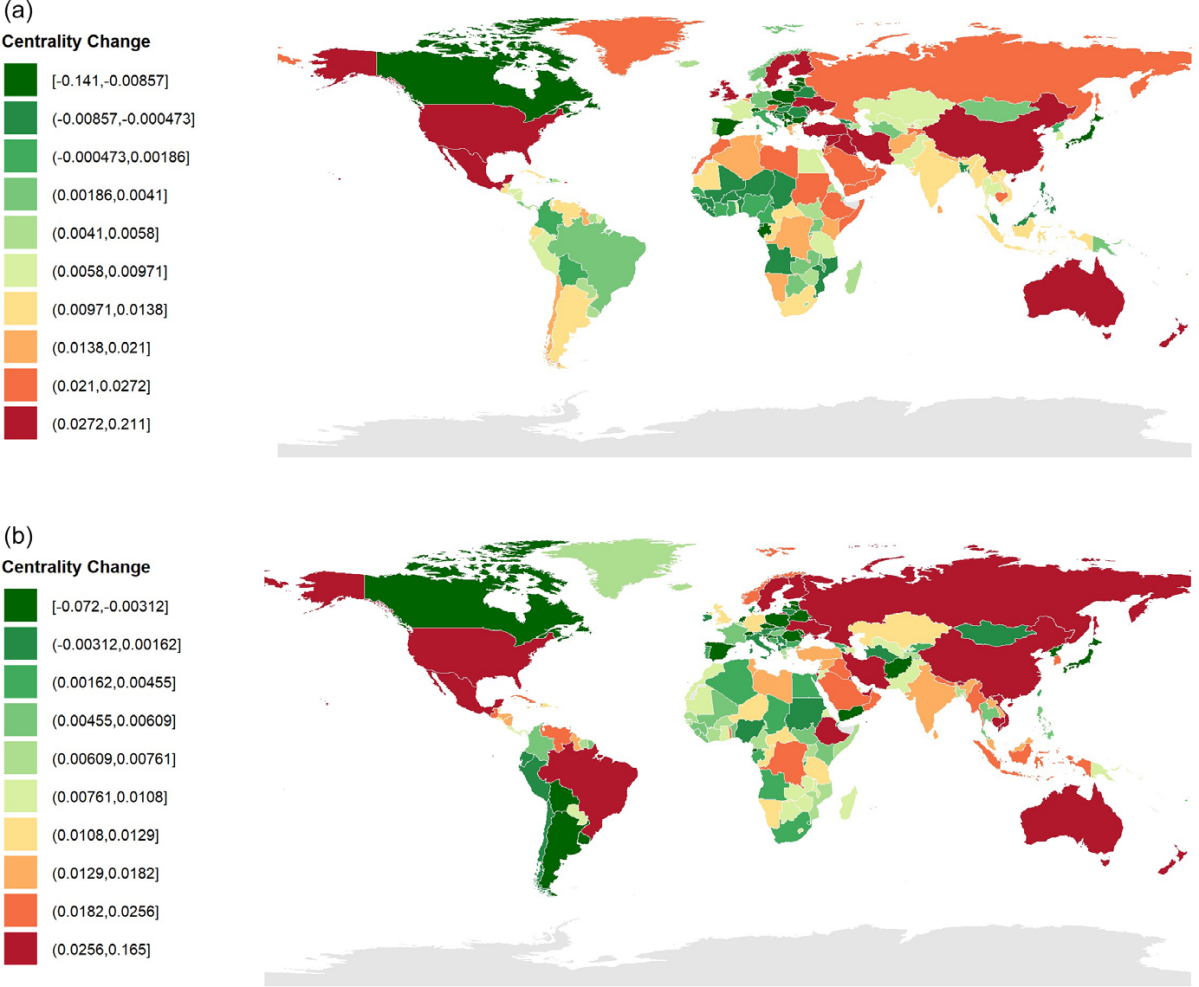
Country-level delta-centrality maps for anorexia nervosa (A) and bipolar disorder (B) in the ecological incidence network. *Note:* Positive delta values indicate that removing a country reduces the estimated strength centrality of the focal disorder in the full network; negative values indicate that removing a country increases it. These values represent influence on the estimated all-countries network, not causal importance or policy priority. Figure 4. long description.

Figure 4 presents the country-level delta-centrality values for anorexia nervosa and bipolar disorder, the two disorders with the highest strength centrality in the full network. Positive delta values indicate that omitting a country reduces the estimated strength centrality of the focal disorder, whereas negative values indicate that omission increases it. For anorexia nervosa, the largest positive delta values were observed in Australia, the United States, Iran, China and Finland. For bipolar disorder, China, Iran, Australia, Brazil and Mauritius had the largest positive delta values. These results indicate influence on the estimated all-countries network and may reflect epidemiological, diagnostic, reporting or health-system differences across countries.

Bootstrap analyses suggested moderate precision for edge estimates and acceptable but limited stability for strength centrality. The case-dropping CS coefficient for strength was 0.28, indicating that the ranking of central nodes should be interpreted cautiously. For this reason, we emphasize strength centrality in the main text and treat the relative ordering of nodes as descriptive rather than definitive (Supplementary Figures S1 and S2).

## Discussion

This cross-sectional ecological study examined how age-standardized incidence rates of 10 mental disorders and the SDI covary across 204 countries. Two main findings emerged. First, anorexia nervosa and bipolar disorder showed the highest strength centrality, meaning that they had the greatest total absolute connectedness with other nodes in the estimated ecological network. Second, a case-deletion analysis revealed that several countries including Australia, the United States, Iran, China and Finland disproportionately influenced the estimated centrality of these focal disorders. Taken together, these findings are descriptive and hypothesis-generating; they do not imply individual-level comorbidity, causal effects or direct policy priorities.

The prominence of anorexia nervosa in the network is not a reflection of its global incidence burden. As shown in Table 1, anorexia nervosa had the lowest age-standardized incidence rate among the included disorders, yet it had the highest strength centrality. This apparent paradox is explained by the nature of network analysis: centrality reflects how strongly a node is conditionally connected to other nodes after adjusting for all other variables, whereas incidence reflects the frequency of new cases. Thus, a relatively uncommon disorder can appear central if its cross-country incidence pattern broadly covaries with other disorders. From a hypothesis-generating perspective, this suggests that country-level factors affecting the recorded incidence of anorexia nervosa, including diagnostic practices, cultural attitudes toward eating disorders, healthcare-system characteristics and case detection, may also be related to broader cross-national patterns in the incidence of other mental disorders, even if the absolute number of anorexia nervosa cases is low. This does not mean that anorexia nervosa is clinically more important than major depressive disorder, but rather that it may be a sensitive marker of broader cross-national variation in mental disorder incidence.

These findings can be interpreted alongside individual-level psychopathology research showing that eating disorders frequently co-occur with other psychiatric disorders, including mood disorders. General population and clinical studies have reported substantial psychiatric comorbidity among people with eating disorders and reviews specifically note comorbidity between eating disorders and mood disorders, including bipolar disorder (McElroy et al., 2006; Hudson et al., 2007; Godart et al., 2015; Keski-Rahkonen and Mustelin, 2016). Although the present study does not measure within-person comorbidity, the central position of anorexia nervosa suggests that country-level eating-disorder incidence may be embedded within broader cross-national patterns of mental disorder incidence. This interpretation is consistent with network approaches to psychopathology, which emphasize connected patterns among mental health problems while requiring caution when moving between levels of analysis (Borsboom and Cramer, 2013; Fried et al., 2017; Robinaugh et al., 2020).

At the same time, the negative conditional edge between anorexia nervosa and bipolar disorder demonstrates why ecological findings should not be equated with clinical comorbidity. Individual-level studies can report comorbidity between eating disorders and bipolar or other mood disorders, whereas an ecological conditional association may become inverse after adjustment for other disorder rates and SDI (Morgenstern, 1995; Roux, 2002; McElroy et al., 2006; Godart et al., 2015). This pattern may reflect cross-national differences in diagnostic practices, case detection, service access, coding and reporting systems or GBD modeling assumptions rather than a true inverse individual-level relationship. These explanations remain speculative and should be tested in future cross-national studies that combine individual-level diagnostic data with country-level contextual measures.

Turning to the second objective, the country-deletion delta-centrality analysis showed that the estimated connectedness of anorexia nervosa and bipolar disorder was especially sensitive to the inclusion of a small group of countries. For anorexia nervosa, the largest positive delta values were observed for Australia, the United States, Iran, China and Finland; for bipolar disorder, they were observed for China, Iran, Australia, Brazil and Mauritius. These findings indicate that the incidence profiles of these countries contribute disproportionately to the full-sample ecological network estimate. They should not be interpreted as evidence that these countries are causal drivers of global mental health patterns, or that the focal disorders are clinically more central within those countries. Rather, the findings identify settings that may be informative for future comparative studies examining whether diagnostic practices, service access, reporting systems, cultural factors or other contextual variables shape cross-national incidence patterns.

The practical value of this study is primarily heuristic. It offers a descriptive, global-scale map of how mental disorder incidence rates covary across countries, and it identifies specific disorders (anorexia nervosa and bipolar disorder) and specific countries that warrant closer attention in future research. The results are not prescriptive. They should not be used to prioritize clinical interventions, allocate global health funding or draw causal conclusions. Instead, they are best used to generate hypotheses for multilevel studies that combine country-level factors (e.g., healthcare expenditure, diagnostic guidelines, cultural attitudes) with individual-level data to disentangle ecological from person-level associations. Any policy-relevant interpretation would require triangulation with longitudinal, individual-level and intervention-based evidence.

## Strengths and limitations

This study has several strengths. First, it used broad global coverage from GBD 2023, allowing a standardized comparison of country-level age-standardized incidence rates across 204 countries and territories. Second, the ecological network approach provided a compact way to describe conditional associations among multiple disorder categories and SDI within a single analytic framework. Third, the study extended disorder-level analysis by using a case-deletion delta-centrality approach to examine how individual countries influenced the estimated network structure.

Several limitations should also be emphasized. The most important limitation is the ecological, cross-sectional design. All analyses were based on country-level modeled incidence estimates, and no individual-level data were available. As a result, the findings cannot be interpreted as individual-level comorbidity and cannot support causal inference. Second, the estimates are derived from the GBD modeling framework and remain subject to uncertainty related to data availability, case definitions and model assumptions. The present analysis did not propagate GBD uncertainty intervals into edge and centrality estimation. Future work could address this by repeatedly sampling from published uncertainty intervals and re-estimating the network to obtain uncertainty-aware distributions for the main statistics. Third, each country contributed equally regardless of population size. This is appropriate for a country-level ecological comparison, but a population-weighted analysis might produce a different network structure, particularly for highly prevalent conditions. Fourth, SDI was the only contextual variable included; other potentially relevant factors, such as inequality, stigma, diagnostic capacity, service coverage and policy context, were not modeled.

Finally, strength centrality stability was acceptable but modest, with a case-dropping bootstrap CS coefficient of 0.28. Therefore, the relative ranking of nodes should be interpreted cautiously, and small differences in centrality values should not be overinterpreted. We did not conduct additional population-weighted, regional or alternative-specification sensitivity analyses in the current revision. These analyses may be informative, but they would not all address the same estimand as the present study. In particular, population weighting would shift the focus from country-level ecological structure to a population-weighted global structure, while regional network estimation would involve substantially smaller samples and potentially less stable estimates for an 11-node regularized model. These extensions are therefore important directions for future research rather than direct substitutes for the present analysis.

## Conclusions

By estimating an ecological network of country-level age-standardized incidence rates for 10 mental disorders and SDI, this study provides a descriptive overview of how modeled incidence rates covary across 204 countries and territories. Anorexia nervosa and bipolar disorder showed the highest strength centrality, and some countries had a relatively large influence on these estimates in case-deletion analyses. These findings are exploratory and hypothesis-generating. They should be used to inform future individual-level, longitudinal and comparative research rather than to infer causality, clinical priority or direct policy priority.

## Supporting information

10.1017/gmh.2026.10244.sm001 Balooch Hasankhani et al. supplementary material Balooch Hasankhani et al. supplementary material

## Data Availability

The underlying country-level estimates used in this study are publicly available from the Institute for Health Metrics and Evaluation Global Burden of Disease 2023 Results Tool. Derived analytic files and R code used for the present network analyses are available from the corresponding author upon reasonable request.

## Supplementary material

The supplementary material for this article can be found at http://doi.org/10.1017/gmh.2026.10244.

## Long descriptions

### Table 1. Long description

The table contains three columns: Cause, Number, and Age-standardized rates per 100,000 population with 95 percent confidence intervals.

* Mental disorders: 400,412,888 cases; rate of 4,862.84.

* Anorexia nervosa: 1,268,428 cases; rate of 16.47.

* Anxiety disorders: 55,244,318 cases; rate of 684.28.

* Attention-deficit/hyperactivity disorder: 4,226,677 cases; rate of 59.91.

* Autism spectrum disorders: 1,197,423 cases; rate of 19.64.

* Bipolar disorder: 2,505,597 cases; rate of 30.47.

* Bulimia nervosa: 16,957,112 cases; rate of 219.08.

* Conduct disorder: 13,625,473 cases; rate of 182.72.

* Dysthymia: 14,094,987 cases; rate of 165.73.

* Major depressive disorder: 289,610,679 cases; rate of 3,464.17.

* Schizophrenia: 1,682,194 cases; rate of 20.37.

Navigate back to Table 1..

### Figure 1. Long description

A network graph with 11 circular nodes. Node 1 is the central hub, connected to almost all other nodes.

* Central Connections: Node 1 (Anorexia nervosa) has strong green edges to Node 2 (Anxiety disorders), Node 7 (Conduct disorder), and Node 6 (Bulimia nervosa). It has a strong red edge to Node 5 (Bipolar disorder).

* Strongest Association: The thickest green edge in the network connects Node 5 (Bipolar disorder) and Node 6 (Bulimia nervosa).

* S D I Node: Node 11 (S D I) is located at the bottom right, showing a strong green edge to Node 6 and a strong red edge to Node 10 (Schizophrenia).

* Peripheral Nodes: Node 4 (Autism spectrum disorders) is at the top left with a faint red edge to Node 2. Node 8 (Dysthymia) is on the far right with a red edge to Node 5.

Legend for Mental Disorders:

1: Anorexia nervosa

2: Anxiety disorders

3: Attention-deficit/hyperactivity disorder

4: Autism spectrum disorders

5: Bipolar disorder

6: Bulimia nervosa

7: Conduct disorder

8: Dysthymia

9: Major depressive disorder

10: Schizophrenia

Legend for Socio-demographic Index:

11: S D I

Navigate back to Figure 1..

### Figure 2. Long description

The graph consists of three vertical panels sharing a common y-axis. The y-axis lists eleven categories from top to bottom: S D I, Schizophrenia, Major depressive disorder, Dysthymia, Conduct disorder, Bulimia nervosa, Bipolar disorder, Autism spectrum disorders, Attention-deficit/hyperactivity disorder, Anxiety disorders, and Anorexia nervosa.

Panel 1: Strength. The x-axis ranges from 0.0 to 2.0. The data line shows a sharp peak at Bipolar disorder (approximately 1.9) and a maximum value at Anorexia nervosa (approximately 2.2). The lowest value is at Autism spectrum disorders (near 0.0).

Panel 2: Closeness. The x-axis ranges from 0.000 to 0.015. The values are relatively stable between 0.010 and 0.015 for most categories, with a significant trough at Autism spectrum disorders (approximately 0.004) and a peak at Anorexia nervosa (approximately 0.017).

Panel 3: Betweenness. The x-axis ranges from 0 to 20. This panel shows high variability with distinct peaks at Dysthymia (approximately 9), Bulimia nervosa (approximately 8), Bipolar disorder (approximately 10), and a maximum peak at Anorexia nervosa (approximately 22). Most other categories, including Autism spectrum disorders and Anxiety disorders, are near 0.

Navigate back to Figure 2..

### Figure 3. Long description

Panel a displays the age-standardized incidence rate for anorexia nervosa. The legend on the left uses a ten-step color gradient where dark green represents the lowest rates [9.13, 12.2] and dark red represents the highest rates [26, 46.9]. Geographically, the highest incidence rates (dark red) are concentrated in Western Europe and Australia. High rates (orange to red) are also visible in North America and parts of South America. The lowest rates (dark green) are concentrated across the African continent and parts of the Middle East.

Panel b displays the age-standardized incidence rate for bipolar disorder. The legend uses a similar ten-step gradient where dark green represents the lowest rates [15.1, 27.2] and dark red represents the highest rates [50.8, 65.1]. In this map, the highest incidence rates (dark red) are concentrated in South America and Mexico. High rates (orange to red) are also prevalent in North America and parts of the Middle East. The lowest rates (dark green) are concentrated in East Asia, specifically China, and parts of Southeast Asia and Africa.

Navigate back to Figure 3..

### Figure 4. Long description

Two vertically stacked world maps labeled a and b. Each map includes a Centrality Change legend on the left with ten color-coded intervals ranging from dark green for negative values to dark red for positive values.

Panel a, anorexia nervosa.

* North America. Canada is dark green while the United States is dark red.

* South America. Brazil and surrounding northern countries are light green, while Argentina is light orange.

* Europe and Asia. Russia is orange. Western Europe shows a mix of light green and red. China is light yellow.

* Africa. Northern and central regions are mostly green, while southern regions like South Africa are orange.

* Oceania. Australia is dark red.

Panel b, bipolar disorder.

* North America. Canada remains dark green, and the United States remains dark red.

* South America. Brazil is now dark red, contrasting with green in neighboring western countries.

* Europe and Asia. Russia and China are both dark red. India is light orange.

* Africa. Central Africa shows more dark green clusters compared to panel a, while northern and southern tips are red and orange.

* Oceania. Australia remains dark red.

Navigate back to Figure 4..
