# Supplementary material for: Global country-level network analysis of mental disorders
Source: Glob Ment Health (Camb). 2026 Jul 20;13:e168. doi: 10.1017/gmh.2026.10244 (PMC13458861; doi:10.1017/gmh.2026.10244)
Supplement: Supplementary file 1 [file S2054425126102441sup001.docx]

Figure S1. Edge-weight stability analysis of the estimated ecological network.

Figure S2. Centrality stability analysis of the estimated ecological network.

Table S1. Weighted adjacency matrix

|  | **V1** | **V2** | **V3** | **V4** | **V5** | **V6** | **V7** | **V8** | **V9** | **V10** | **V11** |
| --- | --- | --- | --- | --- | --- | --- | --- | --- | --- | --- | --- |
| **V1** | 0.00 | 0.31 | 0.27 | 0.00 | -0.39 | 0.38 | 0.30 | -0.20 | 0.00 | -0.27 | 0.13 |
| **V2** | 0.31 | 0.00 | 0.00 | 0.00 | 0.12 | 0.00 | 0.15 | 0.00 | 0.24 | 0.00 | 0.00 |
| **V3** | 0.27 | 0.00 | 0.00 | 0.00 | 0.29 | 0.00 | 0.00 | 0.00 | 0.00 | 0.18 | 0.00 |
| **V4** | 0.00 | 0.00 | 0.00 | 0.00 | 0.00 | 0.00 | 0.00 | -0.05 | 0.00 | 0.00 | 0.00 |
| **V5** | -0.39 | 0.12 | 0.29 | 0.00 | 0.00 | 0.57 | 0.26 | 0.00 | 0.31 | 0.00 | 0.00 |
| **V6** | 0.38 | 0.00 | 0.00 | 0.00 | 0.57 | 0.00 | 0.00 | 0.00 | 0.00 | 0.00 | 0.45 |
| **V7** | 0.30 | 0.15 | 0.00 | 0.00 | 0.26 | 0.00 | 0.00 | 0.00 | 0.00 | -0.16 | 0.00 |
| **V8** | -0.20 | 0.00 | 0.00 | -0.05 | 0.00 | 0.00 | 0.00 | 0.00 | 0.17 | 0.00 | 0.00 |
| **V9** | 0.00 | 0.24 | 0.00 | 0.00 | 0.31 | 0.00 | 0.00 | 0.17 | 0.00 | 0.00 | 0.00 |
| **V10** | -0.27 | 0.00 | 0.18 | 0.00 | 0.00 | 0.00 | -0.16 | 0.00 | 0.00 | 0.00 | -0.35 |
| **V11** | 0.13 | 0.00 | 0.00 | 0.00 | 0.00 | 0.45 | 0.00 | 0.00 | 0.00 | -0.35 | 0.00 |
| **V1** Anorexia nervosa, **V2** Anxiety disorders, **V3** Attention-deficit/hyperactivity disorder, **V4** Autism spectrum disorders, **V5** Bipolar disorder, **V6** Bulimia nervosa, **V7** Conduct disorder, **V8** Dysthymia, **V9** Major depressive disorder, **V10** Schizophrenia, **V11** Socio-demographic Index | | | | | | | | | | | |
