# Supplementary material for: Global country-level network analysis of mental disorders
Source: Glob Ment Health (Camb). 2026 Jul 20;13:e168. doi: 10.1017/gmh.2026.10244 (PMC13458861; doi:10.1017/gmh.2026.10244)
Supplement: Peer review history

# Reviewer report: Review: Global country-level network analysis of mental disorders — R0/PR2

**DOI:** 10.1017/gmh.2026.10244.pr2

**Authors:** Herbert Ainamani

**Affiliations:** Psychiatry , Kabale University School of Medicine, Uganda

Overall Assessment

I would like to commend the authors for this well-conducted and insightful study. The paper presents an innovative global country-level network analysis of mental disorders, using Global Burden of Disease (GBD) 2023 data to explore cross-country patterns in the incidence of ten mental disorders. The study is timely and contributes global mental health literature. Additionally, the findings that anorexia nervosa and bipolar disorders were central nodes in the global incidence network offer interesting insights that may stimulate future research on cross-national determinants of mental health patterns. However, several methodological clarifications and interpretational issues should be addressed to strengthen the manuscript and improve its clarity and policy relevance.

Specif comments

Abstract

1. The statement that “anorexia nervosa and bipolar disorder emerged as the most central conditions” may be unclear for readers unfamiliar with network analysis. The authors may consider clarifying what centrality represents in this ecological network context

2. The abstract notes that several countries “showed strong influence on disorder centrality.” It would be helpful to clarify what this means for good readability.

3. The manuscript cites the GBD 2021 estimate that 13.9% of the global population is affected by mental disorders. Since the study uses GBD 2023 data, it would strengthen the introduction to reference the most recent global burden estimates if available.

4. The text correctly notes that ecological networks reflect associations between country-level incidence rates. However, it may be helpful to more explicitly emphasize the distinction between ecological co-variation and individual-level comorbidity, to prevent misinterpretation.

5. The authors includes Major ten mental disorders, but it is not clear why these specific conditions were selected as “major” They should clarify whether these represent all mental disorders with incidence estimates in GBD 2023, or whether other mental health conditions were excluded.

6. The authors chose a Mixed Graphical Model (MGM) because the Kolmogorov–Smirnov tests indicated non-normal distributions. Since all variables appear to be continuous incidence rates, it would be helpful to clarify whether any categorical variables were present or whether the MGM was used purely as a semiparametric approach for non-normal data.

7. The manuscript briefly mentions negative conditional associations between disorders. It would be helpful to clarify whether these edges are statistically meaningful or relatively weak, and whether their interpretation should be approached cautiously given regularization.

8. Interpretation of centrality values: Anorexia nervosa was identified as the most central node despite having the lowest global incidence rate. A short explanation of how network centrality can differ from prevalence or incidence magnitude would improve interpretability.

9. The correlation stability coefficient for node strength centrality is 0.28, which indicates acceptable but relatively modest stability. The authors may wish to emphasize this point more clearly when interpreting the centrality rankings.

10. The discussion suggests that anorexia nervosa and bipolar disorder may represent important nodes in the global mental health network. It would be useful to emphasize that this reflects statistical centrality within an ecological network, rather than implying clinical priority or causal influence.

11. The interpretation of countries with high delta centrality (e.g., Australia, the United States, Iran, and China) could be expanded slightly. These patterns may reflect data availability, diagnostic practices, or health system differences, rather than purely epidemiological differences.

12. The authors note that all countries contribute equally to the analysis regardless of population size. It may be useful to briefly discuss whether population-weighted sensitivity analyses might produce different network structures.

13. The study does not incorporate the uncertainty intervals associated with GBD estimates. The authors might consider discussing whether future work could propagate this uncertainty into network estimation.

14. The figures are informative but may be difficult to interpret for readers unfamiliar with network analysis. Consider expanding the figure legends to briefly explain:

• what node size represents

• what edge thickness indicates

• the meaning of positive vs negative edges

• the interpretation of delta centrality maps.

In general, the manuscript presents an interesting application of network analysis to examine cross-country patterns in the incidence of mental disorders using Global Burden of Disease 2023 data across 204 countries and territories.

# Reviewer report: Review: Global country-level network analysis of mental disorders — R0/PR3

**DOI:** 10.1017/gmh.2026.10244.pr3

**Authors:** Prodyut Roy

**Affiliations:** Educational Psychology, Independent Scholar, Bangladesh

Overall comments:

While this manuscript shows potential, it is not yet suitable for publication in its current form. I encourage the authors to carefully address the comments and suggestions provided, which will help improve the overall quality of the manuscript.

Introduction:

You have explained gaps in knowledge in a structured way. I would like to request that you include a section on the significance of this study at the end of the introduction section. It would be great if you could include the theoretical or conceptual framework along with the significance of this study.

Methods

You started with the data source; you did not introduce or explain the participants in this study. Could you please include a description of your participants?

I am curious to know the rationale for selecting 10 major mental disorders. Why is it not 11 or 9? How did you identify your selected mental disorders as major mental disorders? Additionally, I am also keenly interested to know about anxiety disorders; anxiety-based disorder is an umbrella term where several mental disorders fall under this category. Could you please clarify how this category of disorder was conceptualized and utilised in your study, and which specific anxiety-related conditions were included within the scope of your analysis?

Moreover, you did not include the research design. Could you include a section for research design, where a justification for employing it would strengthen the credibility of this research?

Though you used a secondary data source, it is still important to explain the ethical considerations for your study because the data contained information about human beings. It is crucial to include the ethical considerations applied to collect data by the Global Burden of Disease Study.

Discussion

For lines 47-59 (page 8 of 16), you have explained the findings of this study. Could you please compare and contextualize this finding with recent relevant studies? In particular, what might explain the co-occurrence of bipolar disorder and anorexia nervosa? I raise this question because, as a clinical psychologist, I am especially interested in understanding the basis of this comorbidity. Across my 11 years of clinical experience, I have not encountered clients presenting with both conditions simultaneously. A brief discussion of possible explanatory pathways would strengthen the manuscript and offer useful insight to readers, clinicians, and future researchers.

For the consecutive paragraphs, you discussed functionality of psychopathology networks, granularity networks, network framework to an ecological level, and ecological patterns; which are irrelevant for the discussion sections. Methodology will be the perfect section to explain these issues. I would recommend revamping the discussion sections following the critical discussion of the findings of this study, not the methodological (e.g., data analysis tools or procedures). Readers are interested in knowing the author’s voice regarding the findings.

# Reviewer report: Review: Global country-level network analysis of mental disorders — R0/PR4

**DOI:** 10.1017/gmh.2026.10244.pr4

Thank you for the opportunity to review this manuscript. This is a timely and ambitious paper that uses GBD 2023 country-level incidence estimates to examine the ecological network structure of 10 mental disorders across 204 countries and territories. The manuscript has several strengths, including the originality of the question, the broad global scope, the effort to incorporate SDI, and the authors’ repeated acknowledgment that the analysis is descriptive and hypothesis-generating rather than causal. I also appreciated the attempt to move beyond disorder centrality alone and examine country-level influence through the delta-centrality approach. Overall, I found the paper interesting and potentially valuable.

That said, I think the manuscript requires substantial revision before it is ready for publication. My main concerns relate to conceptual framing, methodological justification, interpretation of centrality and stability, and some internal inconsistencies in the presentation of the results.

Major Comments

1) The distinction between ecological association and individual-level comorbidity needs to be tightened throughout the manuscript. The paper does acknowledge that this is an ecological study based on country-level incidence rates rather than individual-level psychiatric data, and that causal or individual comorbidity inferences should not be made. However, the manuscript still uses language such as “comorbidity network,” including in Figure 1, and at several points the framing drifts back toward interpretations that sound closer to individual-level disorder comorbidity than to cross-country co-variation in modeled incidence estimates. I would strongly encourage the authors to revise the language throughout so that the framing remains consistently ecological. This is important not only stylistically, but conceptually, to avoid ecological fallacy and to keep the claims aligned with the study design.

2) The rationale for the modeling approach should be explained more clearly. The Methods state that the authors tested normality, found significant deviations, and therefore used a mixed graphical model with LASSO regularization and EAIC-based tuning. However, all variables appear to be continuous country-level measures, and the manuscript would benefit from a fuller explanation of why this specific model was preferable here, how the estimated edges should be interpreted, and whether those edges can be described straightforwardly as partial correlations in this setting. A brief justification of the analytic choices and, ideally, at least one sensitivity analysis using an alternative reasonable specification would strengthen the paper considerably.

3) The interpretation of centrality results should be more cautious. The manuscript places substantial interpretive weight on the centrality findings, especially the prominence of anorexia nervosa and bipolar disorder. However, the reported strength centrality stability is limited, with a CS coefficient of 0.28, which suggests that the ranking of central nodes should be interpreted cautiously. I would suggest focusing the primary interpretation on strength centrality and being more restrained in discussing closeness and betweenness unless the authors can provide a stronger justification for emphasizing those metrics. Relatedly, the abstract, impact statement, and discussion currently read somewhat more confidently than the stability results seem to warrant.

4) There is an important internal inconsistency in the Results that should be corrected. The manuscript states that anorexia nervosa had relatively strong positive associations with bipolar disorder, bulimia nervosa, and schizophrenia. However, the Results later note that one of the weak negative edges was between anorexia nervosa and bipolar disorder, and the supplementary weighted adjacency matrix also lists the anorexia nervosa–bipolar disorder edge as negative (-0.39). This inconsistency affects one of the paper’s central substantive interpretations and should be carefully checked and corrected throughout the Results, Discussion, figure labels, and any supplementary material.

5) The robustness of the findings would be stronger with at least one or two empirical sensitivity checks. I appreciated that the limitations section is candid and thoughtful. In particular, the authors appropriately note that the study is based on modeled GBD estimates, does not propagate uncertainty intervals into the network analysis, weights all countries equally regardless of population size, and includes only SDI as a contextual variable. These are important limitations. At the same time, because these issues are so central to the interpretation of the findings, I think the manuscript would be much stronger if at least some were addressed analytically rather than only narratively. For example, a population-weighted analysis, a regional sensitivity analysis, or a comparison with an alternative network specification would help readers assess how robust the main findings actually are.

6) The implications section would benefit from more restraint. The discussion is appropriately careful in several places, but some statements still edge toward policy or intervention relevance more strongly than the design supports. In particular, language suggesting “priority settings” or “actionable insights” may be too strong for a cross-sectional ecological network based on modeled incidence estimates with modest centrality stability. I would encourage the authors to keep the paper’s contribution centered on descriptive patterning and hypothesis generation, rather than implying direct policy prioritization from the current results.

Minor Comments

1) Please clarify the rationale for selecting these 10 disorders. Given the paper’s global framing, readers may wonder why these specific disorders were included and others were not. A brief justification would improve transparency.

2) It would help to unpack the distinction between centrality and burden more explicitly. Table 1 shows that anorexia nervosa had the lowest age-standardized incidence rate, yet it emerged as the most central node. That is an interesting finding, but it could easily be misread. A brief explanation clarifying that centrality does not equate to burden, prevalence, or policy priority would help readers interpret the results more appropriately.

3) The data availability statement should be revised for consistency. The manuscript states that the analysis used publicly available GBD 2023 data, yet the data availability statement says the datasets are available from the corresponding author upon reasonable request. This should be reconciled. It may be more accurate to state that the underlying data are publicly available from GBD/IHME and to specify separately whether any derived analytic files or code are available on request.

# Editor report: Recommendation: Global country-level network analysis of mental disorders — R0/PR5

**DOI:** 10.1017/gmh.2026.10244.pr5

**Affiliations:** School of Public Health and Preventive Medicine, Monash University Faculty of Medicine Nursing and Health Sciences, Australia

Please address all the revisions suggested by the reviewers.

# Editor report: Decision: Global country-level network analysis of mental disorders — R0/PR6

**DOI:** 10.1017/gmh.2026.10244.pr6

**Affiliations:** London School of Hygiene & Tropical Medicine, United Kingdom of Great Britain and Northern Ireland

No accompanying comment.

# Reviewer report: Review: Global country-level network analysis of mental disorders — R1/PR8

**DOI:** 10.1017/gmh.2026.10244.pr8

**Authors:** Prodyut Roy

**Affiliations:** Educational Psychology, Independent Scholar, Bangladesh

Firstly, thank you to the authors for addressing the comments, though they need to work a little bit to improve the manuscript, particularly the discussion section. Here is my general feedback for the discussion section:

I read the discussion section several times, but I lost interest in it because of the repetition of the results sections. For instance, the second paragraph covers the explanation of the findings, mostly focused on the statistical analysis, where the impact of these findings or the application needs to be explained.

For the first paragraph, in lines 51-52, the authors narrated some comments against the findings; for example, the findings were descriptive and did not imply causal influence and so on. Could you please explain the implications of this finding?

The authors can think outside the box, meaning expanding the search scope to include the mental health literature. Let me give an example, instead of confining within the statistical explanation, authors can validate and compare the findings with existing literature. Eating disorders and mood disorders are found as comorbid disorders, which the literature highlights as a finding; the authors can discuss it.

What the delta-centrality analysis or other statistical analysis found needs to expalin them in the results section; the discussion section demands to explain the findings, not the meaning of the proposition of the delta-centrality analysis. Please claim your proposition with proper citation(s). Please introduce one concept in one paragraph, introducing multiple concepts in a paragraph creating confusion among readers.

The last paragraph of this discussion should be placed under strengths and limitations.

Lastly, please revisit the purpose of this study; somehow, the discussion sections misalign with the aim of this study. For example, one of the practical values of this study is hypothesis-generating. This concept suddenly pops up in the last paragraph of the discussion section.

The discussion section did not discuss the second objective of this study, how the centrality of the most connected disorders varies from country to country.

I strongly suggest revisiting the purpose of this study and revamping the discussion section.

Thank you.

# Reviewer report: Review: Global country-level network analysis of mental disorders — R1/PR9

**DOI:** 10.1017/gmh.2026.10244.pr9

**Authors:** Herbert Ainamani

**Affiliations:** Psychiatry , Kabale University School of Medicine, Uganda

The authors have responded to all my comments and I have no reservation

# Editor report: Recommendation: Global country-level network analysis of mental disorders — R1/PR10

**DOI:** 10.1017/gmh.2026.10244.pr10

**Affiliations:** School of Public Health and Preventive Medicine, Monash University Faculty of Medicine Nursing and Health Sciences, Australia

Thank you for submitting the revised paper. Although, the paper is improved, the reviewer and I are still concerned about your account of the purpose of the study and the discussion of the findings.

I am pleased to offer one further chance to address these concerns and to rewrite the discussion and bring the paper to a publishable standard.

# Editor report: Decision: Global country-level network analysis of mental disorders — R1/PR11

**DOI:** 10.1017/gmh.2026.10244.pr11

**Affiliations:** London School of Hygiene & Tropical Medicine, United Kingdom of Great Britain and Northern Ireland

No accompanying comment.

# Reviewer report: Review: Global country-level network analysis of mental disorders — R2/PR13

**DOI:** 10.1017/gmh.2026.10244.pr13

**Authors:** Prodyut Roy

**Affiliations:** Educational Psychology, Independent Scholar, Bangladesh

Congratulations.

# Editor report: Recommendation: Global country-level network analysis of mental disorders — R2/PR14

**DOI:** 10.1017/gmh.2026.10244.pr14

**Affiliations:** School of Public Health and Preventive Medicine, Monash University Faculty of Medicine Nursing and Health Sciences, Australia

Thank you for submitting the revised paper, which I am pleased to accept.

# Editor report: Decision: Global country-level network analysis of mental disorders — R2/PR15

**DOI:** 10.1017/gmh.2026.10244.pr15

**Affiliations:** London School of Hygiene & Tropical Medicine, United Kingdom of Great Britain and Northern Ireland

No accompanying comment.
